# Efficacy of Manipulative Acupuncture Therapy Monitored by LSCI Technology in Patients with Severe Bell's Palsy: A Randomized Controlled Trial

**DOI:** 10.1155/2020/6531743

**Published:** 2020-12-16

**Authors:** Weizheng Zhong, Haibo Yu, Xiaodan Rao, Jianhuang Wu, Yanhua Gou, Han Cui, Xingxian Huang, Ling Wang

**Affiliations:** ^1^Shenzhen Traditional Chinese Medicine Hospital, Shenzhen 518033, Guangdong, China; ^2^Shenzhen Institutes of Advanced Technology, Chinese Academy of Sciences, Shenzhen 518055, China

## Abstract

To determine if the effect of manipulative acupuncture monitored by laser speckle contrast imaging (LSCI) can improve facial blood perfusion in patients with severe Bell's palsy. This randomized, single-blind, controlled trial included 120 newly diagnosed patients (within 14 days) with severe Bell's palsy (House–Brackmann grading system (HBGS) ≥ grade IV). The patients were randomized (1 : 1) to receive either acupoints acupuncture combined with manipulations of twirling, lifting, and thrusting treatments (manipulative acupuncture) or acupoints acupuncture therapy alone (simple acupuncture). These treatments consisted of a total of 24 sessions, three times per week, and each treatment lasted for 30 min. Following 8 weeks of treatment and 6 months after the initial onset of facial palsy, facial nerve functioning was scored (HBGS) and clinical efficacy was measured. The patients' facial blood perfusion significantly improved following manipulative acupuncture assisted by LSCI compared with that at baseline (*P* < 0.01). At the conclusion of the 8-week treatment, both groups showed improvement; however, the recovery rate was significantly different (manipulative acupuncture 53.3% vs. simple acupuncture 33.9%, *P* < 0.05). Follow-up analysis at 6 months after the onset of facial palsy revealed a significantly higher recovery rate (91.7% vs. 78.0%; *P* < 0.05). In addition, the number of treatments in the observation group was less than that in the simple acupuncture therapy group (*P* < 0.05). Compared with simple acupuncture therapy, manipulative acupuncture therapy led to a more significant recovery rate in the treatment of severe Bell's palsy and required a shorter course of treatment. This trial was registered with ChiCTR1800019463.

## 1. Introduction

Bell's palsy is an acute peripheral facial nerve palsy of unknown cause and accounts for 50% of all cases of facial nerve palsy [1]. The annual incidence rate is between 13 and 34 cases per 100,000 individuals [2]. Bell's palsy is more prevalent in females than in males. Under natural conditions without any treatment, 94% of patients with incomplete lesions returned to normal, whereas only 60% of those with clinically complete lesions exhibited complete recovery [1].

Although the etiology of Bell's palsy remains unclear, the potential causes of Bell's palsy include viral infections such as herpes simplex virus 1, rheumatic swelling, ischemia, immunological reasons, trauma to the nerve, and idiopathic reasons [3]. Bell's palsy occurs due to inflammation of the facial nerve in the narrow fallopian canal. In patients with Bell's palsy, facial nerve swelling was generally present and was reported in decompression surgery [4, 5]. Edema secondary to herpes simplex virus 1, such as inflammation or ischemia, generally resulted in elevated pressure, leading to further vascular damage. Additionally, continuous ischemia inhibits facial nerve regeneration, resulting in severe facial palsy and a low chance of recovery. Therefore, Bell's palsy is significantly affected by the microcirculation of tissues near the facial nerves. In our previous study, laser speckle contrast imaging (LSCI) was able to detect the asymmetrical distribution of facial skin perfusion in patients with Bell's palsy [6]. Moreover, blood perfusion of acute facial palsy, metabolic activity of the affected facial tissues, and facial nerve function have been shown to be significantly improved by acupuncture [7]. These findings suggest that acupuncture plays a beneficial role in Bell's palsy.

However, all these studies are observational; therefore, the causal relationship has not yet been elucidated. This study was designed as a randomized, single-blind, controlled trial to evaluate whether the treatment of manipulative acupuncture monitored by LSCI technology could improve blood perfusion, enhance the curative effect, and reduce the recovery time of patients with severe Bell's palsy.

## 2. Materials and Methods

### 2.1. Study Design and Participants

This was a single-centre, randomized, single-blind, controlled, parallel assignment study of manipulative acupuncture monitored via LSCI technology versus simple acupuncture for the treatment of patients with severe Bell's palsy.

The study protocol was approved by the Medical Research Ethics Committee of Shenzhen Hospital of Traditional Chinese Medicine. Sample size calculation was dependent on a randomized pilot study of 16 patients with severe facial paralysis before the trial. The pilot study found that the cure rate of manipulative acupuncture under LSCI was 87.5% and that of simple acupuncture treatment was 62.5%. Then, we calculated the sample size of the two groups as 116, with an *α* of 0.05 and statistical power of 0.90. Considering a dropout rate of 10%, the sample size was calculated as 128. Considering the difficulty in enrolling patients with severe paralysis, we finally set the sample size as 120 patients in two groups. Patients were recruited through the Acupuncture Department and the Neurology Department of Shenzhen Traditional Chinese Medicine Hospital in Shenzhen, China, from August 2017 to January 2019. All patients who consented to participate in the study provided informed consent.

Patients aged 18–70 years recently diagnosed (within 14 days) with Bell's palsy were assessed by a single professional neurologist for consideration of inclusion in this study at the screening stage. Each patient was assessed via their House–Brackmann grading system (HBGS) score, and patients who were rated IV–VI were considered for this study. Bell's palsy was defined as a sudden occurrence of unilateral paralysis caused by a lesion on the facial nerves. According to the definition of Bell's facial palsy in the Clinical practice guidelines (2013) [8], Bell's palsy can be diagnosed if patients have the following characteristics and symptoms: (1) acute onset (maximum severity within 72 h); (2) the affected side of the face shows muscle stiffness, numbness, and paralysis; wrinkles on the forehead disappear, and the rima oculi enlarge, exposing the eyeball and increasing the secretion of tears; the nasolabial fold becomes shallower, and the corner of the mouth droops and twists to the contralateral side; and (3) the affected side of the face cannot frown, knit brows, or close the eye completely and cannot show teeth or cheek pouch or whistle properly.

The inclusion criteria were as follows: (1) age 18–70 years; (2) diagnosis of Bell's palsy by a neurologist; (3) HBGS score ≥ grade IV; (4) newly diagnosed (disease duration ≤ 14 days); and (5) willingness to participate in the study and provide informed consent. The exclusion criteria were as follows: (1) face paralysis caused by another disease; (2) diagnosed for more than 14 days; and (3) presence of severe comorbidities that could impact the implementation of the treatment and performance.

### 2.2. Randomization and Masking

Patients were randomly assigned (1 : 1) to receive either manipulative acupuncture under LSCI or simple acupuncture treatment via the use of a randomization scheme. The randomization code was computer-generated at the Shenzhen Traditional Chinese Medicine Hospital. The allocation was concealed until shortly before treatment. All follow-up contact with patients, families, and caregivers was performed by investigators who were unaware of the patients' random assignments and medical treatment management. Outcome assessors and data analysts were also blinded to the treatment allocation until the study database was locked.

### 2.3. Treatments and Management

Study interventions were developed by a consensus of acupuncture experts and per the results of our pilot study [7]. Eligible patients received 24 sessions of acupuncture treatment, three times a week, with each treatment lasting 30 min. The treatment duration was 8 weeks. If an HBGS grade of I was attained before 8 weeks, treatment was ceased. The acupuncture protocol was standardized according to the following order of acupoints: Cuanzhu (BL 2), Yangbai (GB 14), Taiyang (EX-HN 5), Quanliao (SI 18), Dicang (ST 4), Yingxiang (LI 20), and Yifeng (TE 17) on the affected side and Chengjiang (RN 24) and Hegu (LI 4) bilaterally.

Acupuncture was performed by three trained professional acupuncturists. Disposable stainless-steel needles (Suzou Huanqiu Acupuncture and Moxibustion Appliance Co., Ltd.) that were 0.3 mm in diameter and 25 mm or 40 mm in length were used. A standard protocol was followed in the manipulative acupuncture group and simple acupuncture group. A standard protocol was followed for each treatment by all neurologists: The protocol was as follows: (1) inserting the needles subcutaneously downwards into Cuanzhu (BL 2) and Yangbai (GB 14) up to 12.5 mm; (2) inserting the needle perpendicularly into Taiyang (EX-HN 5) and Quanliao (SI 18) up to 12.5 mm; (3) inserting the needle from Dicang (ST 4) toward Jiache (ST 6) up to 35 mm; (4) inserting the needle obliquely into Yingxiang (LI 20) along the nasolabial fold up to 7.5 mm; (5) inserting the needle obliquely outward into Chengjiang (RN 24) up to 7.5 mm; and (6) inserting the needle perpendicularly into Yifeng (TE 17) and Hegu (LI 4) up to 20 mm. In the manipulative acupuncture group, following the insertion of the needles, equal manipulations of twirling, lifting, and thrusting were performed until the blood perfusion was left-right balanced or stable blood flow was maintained, as determined by LSCI. These manipulations were repeated every 10 min, and the needles were removed after 30 min. However, the simple acupuncture group only included acupuncture with no manipulations, and similarly, the retained needles were removed after 30 min.

Trained doctors performed the clinical assessments at baseline, following the 8-week treatment period, and 6 months following the onset of facial palsy. The trained doctors measured and recorded the activity of the facial muscles, H-B degree, and the patients' general neurosensory symptoms, including pain, taste, hearing, tear secretion, and adverse events, which were recorded at every visit and before and after treatment. The information on the most commonly reported adverse events, including pain, fainting, and bruising, was carefully recorded. In terms of pain, we established three categories: light (experienced obvious pain during acupuncture but within the tolerable range and without causing continuous discomfort), moderate (experienced obvious and continuous pain during acupuncture that did not persist for more than 1 h and without any influence on follow-up acupuncture treatment), and severe pain (persistent pain for more than 1 h, leading to withdrawal from acupuncture treatment).

### 2.4. Blood Perfusion Measurement Using LSCI

Blood perfusion of patients in the manipulative acupuncture group was monitored using an LSCI device (SIM BFI-WF System, Wuhan SIM Opto-Technology Co. Ltd., China). The standard operation procedure was used for each measurement, and all the operators were trained. When using this device, the skin of the patients was illuminated by a laser light emitted by the LSCI device. The ratio of blood perfusion at the affected area to that at the normal facial area was calculated; the lower the ratio, the poorer was the blood flow recovery. The central wavelength was 785 nm. The reflected laser light was collected by the lens and imaged on a high-resolution camera, which could be translated to a quantitative measurement of blood flow according to the image analysis [9, 10]. Then, a color-coded image showing the distribution of blood perfusion was generated. Red signified a high level of blood perfusion, and blue represented a low level of blood perfusion. The monitor detector was set 25–30 cm above the patients' face, with a maximum field of view of 14.8 × 14.8 cm so that the entire facial area could be illuminated. The frequency used for the blood perfusion image was 1 s, and the size of the image was 512 × 512 pixels.

Regions with rich blood flow, including the eyelids and cheeks, were selected as regions of interest used to reflect the facial blood perfusion. The lens was placed directly above the patients' face after they had been lying for 5 min with their eyes closed and with the same head posture on a pillow, and then acupuncture was performed. Blood perfusion images of the facial skin were taken and numbered before acupuncture, following acupuncture with manipulation, and after removal of the needles. The duration of the manipulative acupuncture was recorded.

The mean blood perfusion in both sides of the eyelids and cheeks at each week of the study and after 6 months was recorded. The relative blood perfusion rate for the normal side over the impaired side was calculated to reduce the potential residual confounders irrelevant to blood perfusion.

### 2.5. Outcomes

The primary outcome of this study was that the clinical recovery rate was defined based on HBGS after 8 weeks of treatment and 6 months after the onset of facial palsy. The HBGS grade was assessed by two independent professional neurologists who observed facial movement at rest, with a forced smile, with raised eyebrows, and with eyes tightly closed. According to the House–Brackmann facial nerve grading system published in 2013, the sum of the score at the 4th position was divided into six grades, with grade I indicating normal and grade VI indicating total paralysis [8]. The recovery rate was defined as patients with HBGS grade I, and the recovery rate was then calculated. The secondary outcome was the number of treatments required to recover from impaired facial nerve function to grade I.

### 2.6. Statistical Analysis

All participants completed the entire treatment and were included in the analyses of primary and secondary outcomes. Means and standard deviations (±SD) were calculated for continuous variables. We used the *t*-test to compare differences in age and course of disease between the treatment groups. The Wilcoxon test was used to compare the grading of the facial nerve function between patients who underwent manipulative acupuncture under LSCI and those who underwent simple acupuncture because of the unevenness of the variances between the two groups. The paired samples *t*-test was used to compare HBGS changes over time in each group and changes in blood perfusion before and after acupuncture in the manipulative acupuncture group. The chi-square test was used to analyse categorical variables.

All analyses in this study were conducted using SPSS version 16.0 statistical software (SPSS Inc., Chicago, Illinois, USA). A (two-side) *P* value of <0.05 was considered statistically significant.

## 3. Results and Discussion

**Figure 1:**
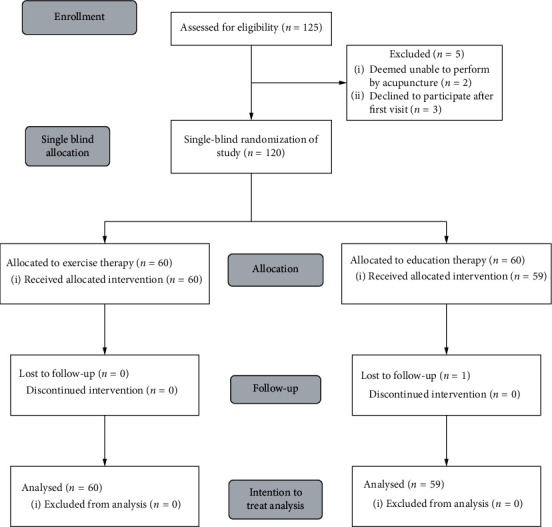
Flow chart for patients' selection.

From August 2017 to January 2019, 125 eligible patients with Bell's palsy were screened for enrolment at Shenzhen Traditional Chinese Medicine Hospital. A total of 120 patients were willing and eligible to attend this study. Among the five excluded patients, two were sensitive to acupuncture pain and were unable to undergo acupuncture treatment and three were unwilling to participate in the study. Overall, 120 patients completed randomization; 60 patients aged 20–69 years (males, 55.0%) underwent manipulative acupuncture treatment (acupoints acupuncture combined with manipulations of twirling, lifting, and thrusting treatments), and 60 patients aged 19–69 years (males, 50.85%) underwent simple acupoints acupuncture treatment (Figure 1). Among them, 43 (72.9%) in the simple acupuncture group and 45 (75.0%) in the manipulative acupuncture under the LSCI group were in the acute stage of facial palsy (*P*=0.904). All participants adhered to the study intervention during the 6-month follow-up. Only one patient withdrew after 2 weeks of treatment in the simple acupuncture treatment group due to personal reasons.

**Figure 2:**
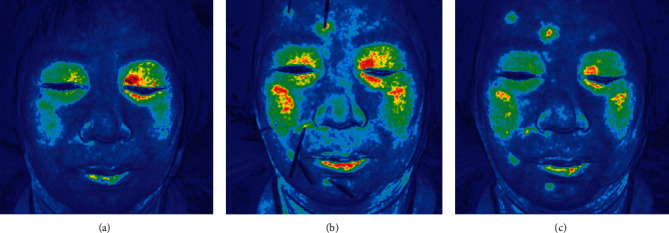
Blood perfusion monitored using LSCI technology in a patient with severe Bell's palsy. (a) Before acupuncture. (b) Acupuncture with manipulation. (c) After removing the needles.

**Figure 3:**
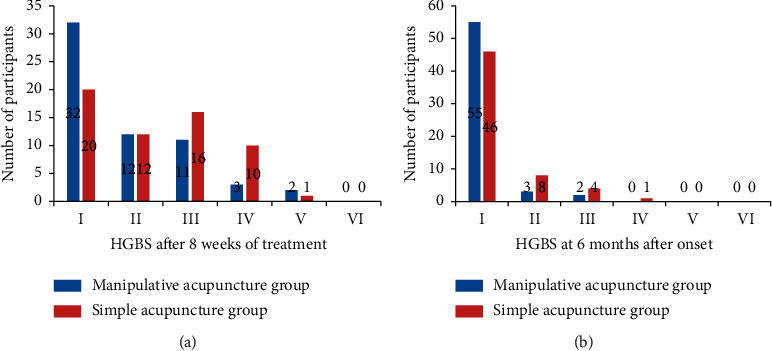
HBGS grade of participants in the two groups after 8 weeks of treatment and at 6 months after onset.

The baseline characteristics of the patients, including sex, age, duration of disease, and HBGS grade, were similar between the two groups (all *P* values >0.05; Table 1). Blood perfusion at both eyelids and cheeks in the manipulative acupuncture group was significantly increased (all *P* values <0.01), the ratio of the affected area to normal face area decreased from 1.35 ± 0.14 to 1.15 ± 0.09 at the eyelids and from 1.29 ± 0.11 to 1.07 ± 0.08 at the cheeks (Table 2 and Figure 2). Manipulative acupuncture significantly improved the facial nerve function HBGS grade of 60 patients. HBGS grades higher than IV at baseline to 5 (8.33%) at 8 weeks and 0 at 6 months were compared with those in the simple acupoints acupuncture group with 11 patients with HBGS grades higher than IV at 8 weeks and 1 at 6 months (all *P* values <0.05, Table 3 and Figure 3). Five patients each in the simple acupuncture and manipulative acupuncture groups presented acute exacerbation during the follow-up period (*P*=0.48).

**Figure 4:**
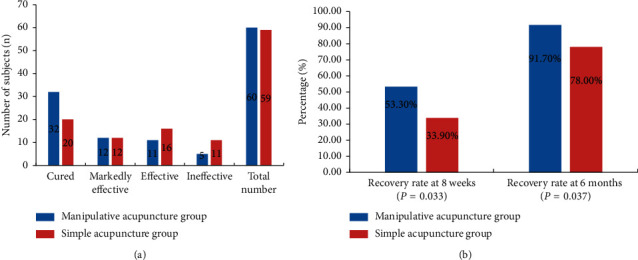
Clinical efficacy (number of participants and recovery rate) of participants in the two treatment groups after treatment.

Following the 8-week treatment, the recovery rate at 6 months was 91.7% in the manipulative acupuncture group, which was nearly 10% higher than that in the simple acupuncture group (81.4%), and the *P* value was 0.033. In addition, the recovery rate at 8 weeks for manipulative acupuncture was 53.3%, which was 20% higher than that for simple acupuncture (33.9%), and the *P* value was 0.037 (Table 3 and Figure 4).

The number of treatments required to recover from impaired facial nerve function to HBGS grade I was 16.75 ± 4.09 in the manipulative acupuncture group, which was significantly less than that in in the simple acupuncture group (19.65 ± 2.96) (*P*=0.008, Table 3).

Bruising was a unique adverse event reported in the study; 18.33% of patients in the manipulative acupuncture group and only 13.56% of patients in the simple acupuncture group reported grade I bruising (*P*=0.477) (Table 4). In addition, 12 patients (20%) in the manipulative acupuncture group and 10 (16.95%) in the simple acupuncture group reported experiencing light pain, and 5 (8.33%) in the manipulative acupuncture group and 3 (5.08%) in the simple acupuncture group experienced moderate pain. No other adverse events were noted in both groups.

## 4. Discussion

This study found that using simple acupoints for both acupuncture and manipulative acupuncture significantly improved the HBGS grade of severe Bell's palsy, resulting in a higher recovery rate at 8 weeks after treatment and at 6 months after the onset of facial palsy. Moreover, fewer treatments were required to recover the impaired facial nerve function to grade I.

A peripheral facial palsy is a common clinical syndrome due to complex causes, including disease, gene, and environmental factors. Although the underlying mechanism is uncertain, evidence suggests that herpes simplex virus activation might be a likely cause of Bell's palsy. Nevertheless, it is difficult to identify whether Bell's palsy is caused by herpes simplex virus as there are no established or widely available methods to confirm the activity of the herpes simplex virus. The pathological changes that occurred in patients with Bell's palsy were primarily facial nerve edema and degeneration caused by compression of the myelin sheath or axon. Glucocorticoids were generally recommended for early and short-term use to eliminate inflammatory edema. However, recently, some researchers have reported that facial neuritis could lead to various blood circulation disorders. Moreover, the recovery of facial nerve function depended on an appropriate blood supply to maintain the nerve environment and nutrition supply [11]. Thus, increasing this blood supply might benefit the facial nerve of patients with facial palsy. However, currently, there is a lack of effective drugs or treatments designed to specifically improve the blood supply of the facial nerve for these patients. Therefore, in this study, we used LSCI technology to monitor the blood perfusion of the facial palsy patients and evaluated whether manipulative acupuncture could significantly improve the blood supply of the facial paralysis side compared with simple acupuncture. The results of this study showed efficacy for manipulative acupuncture as a new treatment method for Bell's palsy, particularly for severe cases. Manipulative acupuncture had significantly improved the facial nerve function and recovery rate at 8 weeks of treatment and at 6 months following the onset of the palsy. Moreover, this study also confirmed the hypothesis that the blood supply might play a critical role in the prognosis of facial palsy.

Acupuncture has been widely used in the treatment of facial paralysis in China and has shown remarkable efficacy for various conditions. However, the underlying mechanism of acupuncture treatment has not yet been explicated. Traditional Chinese medicine believes that acupuncture treatment regulates and maintains the Yin-Yang balance. One of the theoretical functions of Yin and Yang is blood flow. Acupuncture into acupoints did not puncture the facial nerve or the blood vessels; however, it promoted blood circulation. Therefore, the manifestation of needling Deqi is described as the physician's sense of compactness and the patient's sense of soreness, heaviness, and distension during needling. However, these are subjective sensations, which required experienced doctors for the needling process and the patients' careful experience and cooperation. Thus, it is often difficult to obtain satisfactory clinical results. While “blood” is a subjective index, it was successfully evaluated using LSCI technology. Laser speckle technology could capture facial blood perfusion and monitor the changes of blood perfusion dynamically during the whole process of acupuncture. LSCI is important for acupuncturists as they can observe the changes occurring due to this treatment concretely, timely, and as a standardized treatment. This study showed that manipulative acupuncture stimulation significantly improved the blood perfusion in the paralysis area, shortened the recovery time of patients with severe Bell's palsy, and ultimately improved the recovery rate.

### 4.1. Strengths and Limitations of this Study

This was a single-centre, randomized, single-blind, controlled, parallel assignment trial, and a larger-scale trial is required. Although the acupuncturists and patients were not blinded to the data, the evaluators were blinded, which ensured objectivity and accuracy of blood perfusion in the manipulative acupuncture group, which was the main focus of this study. However, our primary outcome was the HBGS grade, which accurately represented the efficacy of the patients at 8 weeks of treatment and 6 months following the onset of facial palsy. Further research is still required, and a large and well-designed trial using more effective acupuncture methods is warranted.

## 5. Conclusions

The results from this parallel controlled study suggested that manipulative acupuncture is an acceptable treatment for patients with severe Bell's palsy to improve facial blood perfusion and facial nerve function via HBGS grade, leading to increased efficacy and recovery rate and requiring fewer treatments to recover from the impaired facial nerve function to HBGS grade I. Therefore, further testing is required with a larger number of patients and participating hospitals.

## Figures and Tables

**Table 1:** Patients' characteristics at baseline.

Characteristics	Acupuncture group (*n* = 59)	Manipulative acupuncture under LSCI group (*n* = 60)	*P* value
Age (years)	19–59	20–69	
Males, *n* (%)	38.47 ± 13.33	42.52 ± 13.47	0.103
Duration of disease (days)	1–13	1–14	0.670

HBGS (*n*)			
IV	51	52	
V	7	6	
VI	1	2	
Acute stage	43 (72.9)	45 (75.0%)	0.904

**Table 2:** Comparison of blood perfusion between before acupuncture and after needle removal in the observation group.

Blood perfusion	Before acupuncture	After acupuncture	*P* value
Blood perfusion ratio at eyelids	1.35 ± 0.14	1.15 ± 0.09	<0.01
Blood perfusion ratio at cheeks	1.29 ± 0.11	1.07 ± 0.08	<0.01

Blood perfusion ratio: blood perfusion at the affected area to that at the normal facial area.

**Table 3:** Primary and secondary outcomes after 8 weeks of treatment and after 6 months after the onset of facial paralysis.

Characteristics	Acupuncture group (*n* = 20)	Manipulative acupuncture under LSCI group (*n* = 32)	*P* value
Recovery rate at 8 weeks (%)	33.9	53.3	0.033
Recovery rate at 6 months (%)	78.0	91.7	0.037
HBGS grade at 8 weeks (*n*)	11	5	0.018
IV	10	3	0.038
V	1	2	
VI	0	0	
HBGS grade at 6 months (*n*)	1	0	
IV	1	0	
V	0	0	
VI	0	0	
Number of treatments at 6 months	19.65 ± 2.96	16.75 ± 4.09	0.008
Acute exacerbation	5 (25.0%)	5 (15.6)	0.48

**Table 4:** Summary of adverse events in the manipulative acupuncture vs. simple acupuncture groups.

Treatment group	Bruising	Light pain	Moderate pain	Severe pain	Fainting
Manipulative acupuncture (*n* = 60)	11 (18.33%)	12 (20%)	5 (8.33%)	0	0
Simple acupuncture (*n* = 59)	8 (13.56%)	10 (16.95%)	3 (5.08%)	0	0

## Data Availability

The data used to support the findings of this study are included within the article.
